# Application of sutureless corneal incision for patients with congenital ectopia lentis - Is it feasible, effective and safe?

**DOI:** 10.7150/ijms.93994

**Published:** 2024-05-30

**Authors:** Yang Sun, Tianhui Chen, Zexu Chen, Wannan Jia, Yan Liu, Zhennan Zhao, Yongxiang Jiang

**Affiliations:** 1 Department of Ophthalmology and Vision Science, Eye and ENT Hospital of Fudan University, Shanghai, China.; 2 NHC Key Laboratory of Myopia (Fudan University), Key Laboratory of Myopia, Chinese Academy of Medical Sciences, and Key Laboratory of Visual Impairment and Restoration of Shanghai, Shanghai, China.

**Keywords:** congenital ectopia lentis, sutureless hydroclosure, corneal sutures, clinical outcomes, suture-related complications

## Abstract

**Purpose:** To compare the clinical outcomes, feasibility, and safety between groups with sutured and sutureless wound closure in congenital ectopia lentis (CEL) patients.

**Methods:** Patients with CEL who received phacoemulsification combined with intrascleral fixation of capsular hook (CH) and implantation of capsular tension ring (CTR) and in-the-bag intraocular lens (IOL) were included in this study.

**Results:** A total of 68 eyes of 34 patients aged 18 years or younger were enrolled in this study. Incisions of 21 patients (34 eyes) did not require sutures while sutures were applied in 21 patients (34 eyes). Postoperative uncorrected distance visual acuity, best corrected distance visual acuity and intraocular pressure measurements were comparable on follow-up visits (P > 0.05). The magnitude of surgically induced astigmatism was significantly greater (P = 0.001) in the suture group (Median: 0.47; IQ: 1.63, 2.97) than in the sutureless group (Median: 0.88; IQ: 0.63, 1.35). No cases of endophthalmitis and retinal detachment were found postoperatively in either group, while suture-related complications were observed in the sutured group, including loose suture with discomfort in 5 (14.71%) eyes, loose suture with mucus infiltration in 3 (8.82%) eyes. In total, 22 sutures (64.71%) of 34 eyes required removal.

**Conclusions:** Sutureless clear corneal incision in CEL patients can achieve satisfactory clinical results comparable to sutured wound closure in terms of the efficacy and safety. Advantages of this approach are the reduced risk of suture-related complications, no need for additional surgery under general anesthesia for suture removal, and less cost.

## Introduction

Congenital ectopia lentis (CEL) is a hereditary bilateral disease characterized by the lens dislocation from its anatomical position due to the laxity of zonule fibers induced by zonular dysplasia.^1^ With an estimated 6.4 cases per 100,000 children, CEL is the second most common reason for lens surgery treated in the pediatric population after congenital cataract.^2^ It typically presents with myopia, astigmatism, resulting in permanent vision loss caused by anisometropic amblyopia in juveniles.^3^, ^4^ Currently, surgical removal of the dislocated lens constitutes one of the most common and effective treatments for correcting the refractive errors and restoring vision loss in CEL patients.

Surgical management for patients with CEL has been improved with ongoing refinement of machines, vitrectors, and intraocular lens (IOL). In recent years, lens capsule stabilizing devices such as capsular tension ring (CTR) and modified capsular tension ring (MCTR) have been recognized as providing better stability for the lens capsule with zonular dialysis, and several studies have demonstrated good clinical efficacy in CEL patients implanted with MCTR.^3^, ^5^, ^6^ However, the implantation of MCTR requires relatively large incision, which is susceptible to complications such as wound leakage and postoperative astigmatic error. In order to reduce the incision size and avoid large conjunctival dissections during surgery, an improvement of sutureless surgical technique using intrascleral fixation of capsular hook (CH) has been introduced by Jin *et al.*^7^, with simpler manipulations of suturing, shortened operation time, and less risk of complications compared with other techniques. By applying this technique, we have identified a decrease in the incidence of retinal detachment, vitreous prolapse, IOL dislocation in CEL cases, whereas the suture-related complications have become one of the most prominent complications, including neovascularization, eye irritation and infections due to the loose or broken sutures, and astigmatism induced by tight sutures that may lead to vision deteriorating and amblyogenic.^8^ In addition, suture removal is required irrespective of its material, which can necessitate general anesthesia or sedation in children and increase medical costs.^9^ Hence, it is necessary to avoid sutures in wound closure in CEL patients to optimize the surgical treatment effect and reduce the suture-related complications.

In pediatric cataract surgery, sutureless hydroclosure approach can achieve stable wound and satisfactory outcomes, which is not inferior to the standard approach.^10^-^12^ However, no study has been done where the wounds were not sutured in CEL patients. We therefore launched a first attempt to perform sutureless surgery in CEL patients who underwent phacoemulsification combined with implantation of CTR and intrascleral fixation of the CH. Herein, we reported our experience of sutureless surgical procedure and conducted a comparative analysis of clinical outcomes, feasibility, and safety between groups with sutured and sutureless wound closure.

## Methods

This retrospective, observational, case series study comprised patients 18 years of age or younger with CEL in Eye and Ear, Nose, and Throat Hospital of Fudan University between January 2021 and March 2023. It was approved by the Human Research Ethics Committee that adhered to the tenets of the Declaration of Helsinki, and the institutional review board approved the study as an extension of our randomized controlled trial (ChiCTR2000039132). This work has been reported in line with the STROCSS criteria.^13^ All the families of the patients were routinely informed about the surgical purpose, management protocol, recovery period, possible complications and postoperative care before surgery, and the written informed consent was taken from the patient's legal guardian preoperatively.

### Patient eligibility

Patients with CEL who received phacoemulsification combined with intrascleral fixation of CH and implantation of CTR and in-the-bag IOL were eligible for inclusion.

The inclusion criteria were as follows: (1) patients diagnosed as congenital ectopia lentis with mild to moderate lens subluxation; (2) patients who were 4 years of age and older and were able to cooperate with preoperative and postoperative examinations; (3) uncorrectable lenticular astigmatism; (4) pupillary blocking glaucoma as a result of the dislocated lens; (5) a high risk of amblyopia progression.

Patients with the following features were excluded from the study: (1) patients with severe subluxation; (2) a history of ocular trauma or other ophthalmic surgery, such as congenital cataract, retinal detachment, epiretinal membrane, or anti-glaucoma surgery; (3) failure to cooperate with optometric measurements and/or the postoperative data were missing.

### Examinations

All enrolled patients underwent a comprehensive preoperative ophthalmologic examination, including uncorrected distance visual acuity (UDVA) (logarithm of the minimal angle of resolution [logMAR]) and best corrected distance visual acuity (BCVA) (logMAR). Visual acuity was determined using an international standard chart at a distance of 5 m, with refractive errors adjusted based on readings from an automatic refractometer and keratometer (ARK-700A; Nidek, Aichi, Japan). Intraocular pressure (IOP) was measured using noncontact tonometry (Canon TX-20, Canon, NY, USA). The severity and degree of lens subluxation was evaluated via slit-lamp examination after mydriasis. Ocular biometric parameters, including axial length (AL), anterior chamber depth (ACD, measured from epithelium to lens), lens thickness (LT), mean keratometry (Km), flat keratometry (Kf), steep keratometry (Ks) and corneal astigmatism were performed with a partial coherence interferometer (IOL Master 700, Carl Zeiss Meditec AG, Jena, Germany), conducted by the same skilled professional under non-cycloplegic conditions.

In all cases, one of the following IOL models was implanted in this study: TECNIS ZCB00 (Johnson & Johnson, New Jersey, USA) and AcrySof SN60WF (Alcon Laboratories Inc., Texas, USA). Both IOLs are made of hydrophobic acrylic and shared the biconvex single-piece asymmetric design with two C haptics. Considering the similar characteristics between these IOLs, the risk of potential bias introduced by the inclusion of eye implanted with different IOLs have been decreased.

### Surgical procedures

All surgeries were performed under general anesthesia by an experienced surgeon (Dr. YX Jiang) with a corneal clear incision of 2.2 mm. After the injection of the ophthalmic viscosurgical device (OVD) into the anterior chamber, a continuous curvilinear capsulorhexis (CCC) with a diameter of 4.0-5.0 mm was made. Temporary capsular retractors (CapsuleCare, Med Devices Lifesciences, India) were used to suspend and stabilize the capsular bag.

The surgical procedures and the method of intrascleral fixation of implantable capsular hooks were identical to that reported in previous publications by Jin *et al.*^14^ The surgeon performed irrigation/aspiration (I/A) under a low vacuum with a reduced bottle height of 60 cm by phacoemulsifer to remove the lens material in children. Next, the pre-loaded CTR (Bausch & Lomb, Rochester, NY) was then implanted into the capsular bag to further stabilize the bag.

The capsular hook was made of 5-0 commodity polypropylene thread and a disposable high-temperature cautery device was applied to form a permanent bend by thermoplasticity. The needle attached to the capsule hook was inserted through the main or side-point incision opposite to the fixation site and guided out through the sclerotomy that penetrated through the ciliary sulcus at the fixation site 1.5 to 2.0 mm posterior to the corneal limbus. An intrascleral pass of the needle shaft of the hook was performed before introducing the hook into the anterior chamber. The needle was passed around the interlamellar sclera with a modified knotless Z-suture technique and the externalized shaft of the hook was then held and pulled by the needle holder or forceps to adjust its tension. A 23-gauge end-gripping microforceps (Grieshaber; Alcon, Fort Worth, TX) was simultaneously used to grip the hook and introduce it into the anterior chamber. The hook was further placed to hold the CCC rim. After implanting the hook to engage the CCC rim, gently released the microforceps. Later, pulled or pushed the externalized shaft to position the capsular bag to further adjust the tension of the hook. The number of hooks was decided by the severity of the zonular defect. Usually, one hook was applied to fixate the capsular bag with a zonular defect of less than six clock hours. In zonulodialysis of more than 6 clock hours and less than 8 clock hours, two hooks were needed to fixate the capsular bag.

After removal of the temporary capsular retractors, a foldable IOL was implanted into the capsular bag. The externalized tip of the hook was trimmed with scissor to flush with the scleral surface. Then, the end of the tip was pushed and buried into the scleral tunnel. The corneal stroma was hydrated with balanced saline solution on a straight cannula in all the wounds. Next, gentle external pressure on corneal apex was applied to assess the watertight seal. If hypotony or shallowing of the anterior chamber was noticed, the corneal incision was closed with 10-0 nylon sutures, and the conjunctiva flap was subsequently closed using 8-0 nylon sutures. In the absence of wound leakage, iris prolapse, or vitreous loss, the corneal incisions were left sutureless. The patients with sutureless corneal wound were classified in the sutureless group and those with sutured corneal incision were put into the sutured group.

### Outcomes

The primary clinical outcomes, assessed centrally at Eye and Ear, Nose, and Throat Hospital of Fudan University, were the difference between the two groups in visual and refractive outcomes, including UDVA, BCVA, IOP measurement, manifest refraction spherical equivalent, manifest refraction sphere and cylinder at the 3-month follow-up visit.

Additionally, secondary clinical outcomes included corneal astigmatism, surgically induced astigmatism and the incidence of complications between groups with sutured and sutureless wound closure.

### Assessment of Corneal Astigmatism and Surgically Induced Astigmatism

The magnitude and the axis of corneal astigmatism were measured using IOL Master 700 (Carl Zeiss Meditec AG, Jena, Germany) at least 5 times immediately after blinking based on the manufacturer's instructions and the average value was used for statistical analysis. We calculated the surgically induced astigmatism (SIA) from the keratometric values obtained preoperatively and postoperatively using the SIA Calculator Version 2.1 developed by Drs Saurabh Sawhney and Aashima Aggarwal (http://www.insighteyeclinic.in/SIA_calculator.php)^15^ according to the vector analysis algorithm.^16^, ^17^ The arithmetic mean of surgically induced astigmatism (M-SIA) and the centroid of surgically induced astigmatism (C-SIA) were assessed by vector analysis and the individual distributions were visualized using the astigmatism double angle plot tool available on the American Society of Cataract and Refractive Surgery website (https://ascrs.org/tools/astigmatism-double-angle-plot-tool).^18^

Subgroup analysis was performed based on the preoperative astigmatism, which was divided into two subgroups according to the differences between the preoperative astigmatism axis (flattest axis) and the incision axis (135 degrees) as follows: Subgroup I, 0 ≤ absolute value of the difference ≤ 45 degrees; Subgroup II, 45 < absolute value of the difference. An incision made at 135 degrees in Group I was an incision made close to the flattest meridian. On the other hand, an incision made at 135 degrees in Group II was an incision made close to the steepest meridian. We then analyzed the SIA in each preoperative astigmatism axis-stratified group (Groups I, II).

### Complications

Intraoperative and postoperative complications considered for analysis were: intraoperative posterior capsule rupture, radial tears in capsulorhexis, vitreous loss, iris prolapse, retinal detachment, dislocation of IOL-capsular bag complex, and endophthalmitis up to last follow-up.

**Figure 1:**
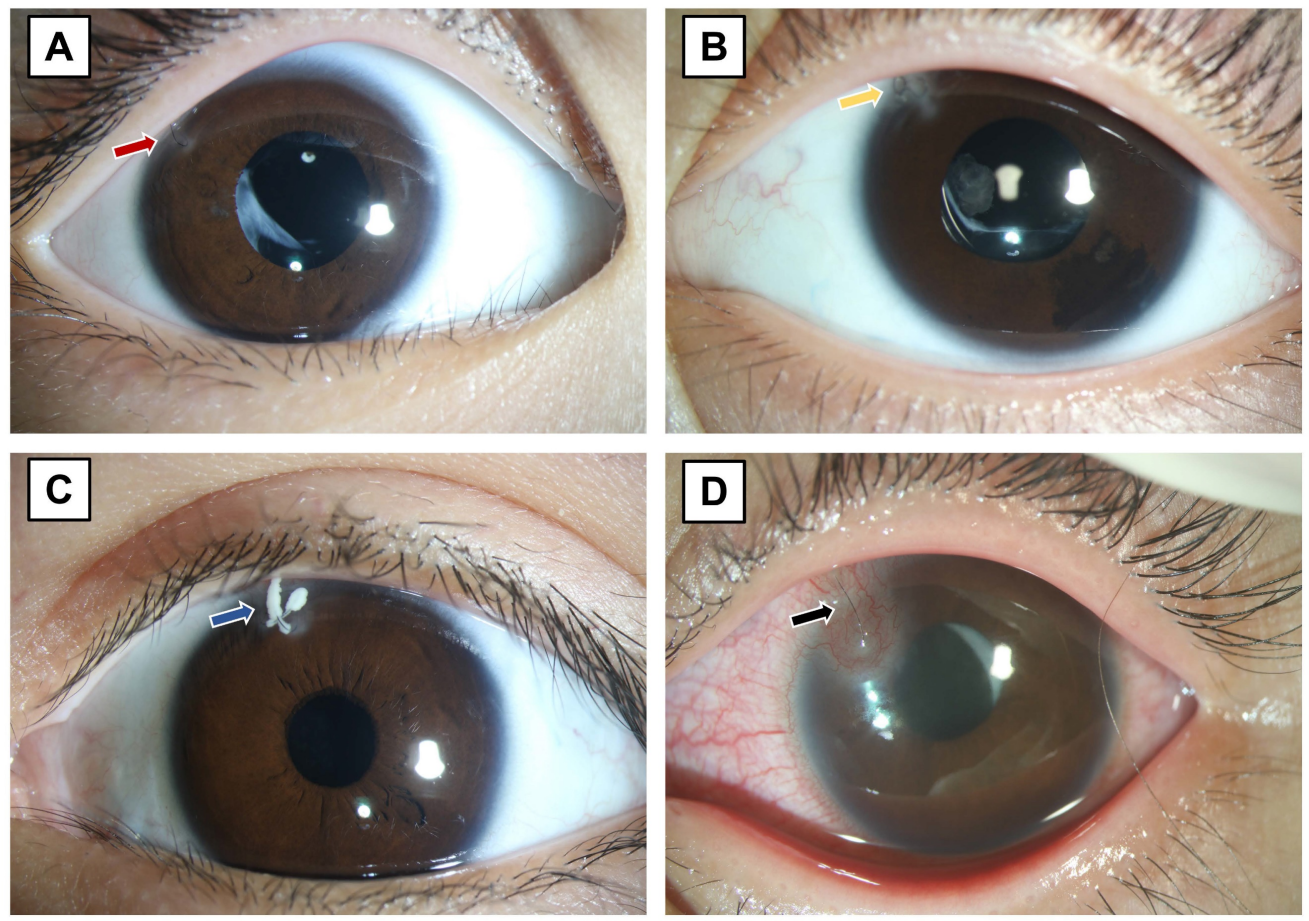
Suture-related complications. (A) Loose sutures (red arrow), (B) breaking of sutures (yellow arrow), (C) loose suture with mucus infiltration (blue arrow), and (D) inflammatory reaction characterized by vascularization around the suture with conjunctival hyperemia (black arrow).

As shown in Figure 1, suture-related complications included loose sutures (Figure 1A), breaking of sutures (Figure 1B), mucus infiltration (Figure 1C), inflammatory reaction characterized by vascularization around the suture with conjunctival hyperemia (Figure 1D), or a combination. The postoperative discomfort was reported by the young patients or their parents, based on their subjective feelings and behavior including rubbing the operated eyes and blinking frequently.

### Statistical analyses

Statistical analyses were performed using SPSS statistical software for Windows (version 22, IBM Corp.). Normality of the data distribution was tested by the Shapiro-Wilk test. Continuous data were shown as the mean ± standard deviation (SD) or median with interquartile range (IQ). Group comparisons between continuous variables were performed using the Student's t-test for parametric variables or Mann-Whitney U test for nonparametric variables. Generalized estimated equation model was used for adjustment of correlation between 2 eyes of a participant. The comparison between preoperative and postoperative data within the same group was analyzed by paired Student's t-test or paired Wilcoxon signed rank test. Categorical variables were analyzed using Chi-square and Fisher's exact tests. Differences were considered statistically significant if P values were less than 0.05.

## Results

### Demographic and ocular biometry characteristics of participants

A total of 68 eyes of 42 patients aged 18 years or younger were included. Preoperative demographics of the study population were summarized in Table 1. There were 12 boys and 9 girls in the sutureless group and 13 boys and 8 girls in the sutured group. The median age of the investigated subjects was 5 years (IQ: 5, 8) ranged between 4 and 10 years and 5 years (IQ: 4, 6) ranged between 4 and 10 years in sutureless and sutured group, respectively. Age and gender did not differ significantly between the two groups (P = 0.124 and P = 0.753). 34 eyes had sutureless wound closure and sutured wound closure, respectively. The median follow-up was 10.82 ± 4.78 months in the sutureless group and 10.47 ± 6.43 months in the sutured group. There were no significant differences in the preoperative biometrics, such as AL (P = 0.598), LT (P = 0.185), and keratometry between the two groups.

### Visual and refractive outcomes in the sutureless and sutured group

**Figure 2:**
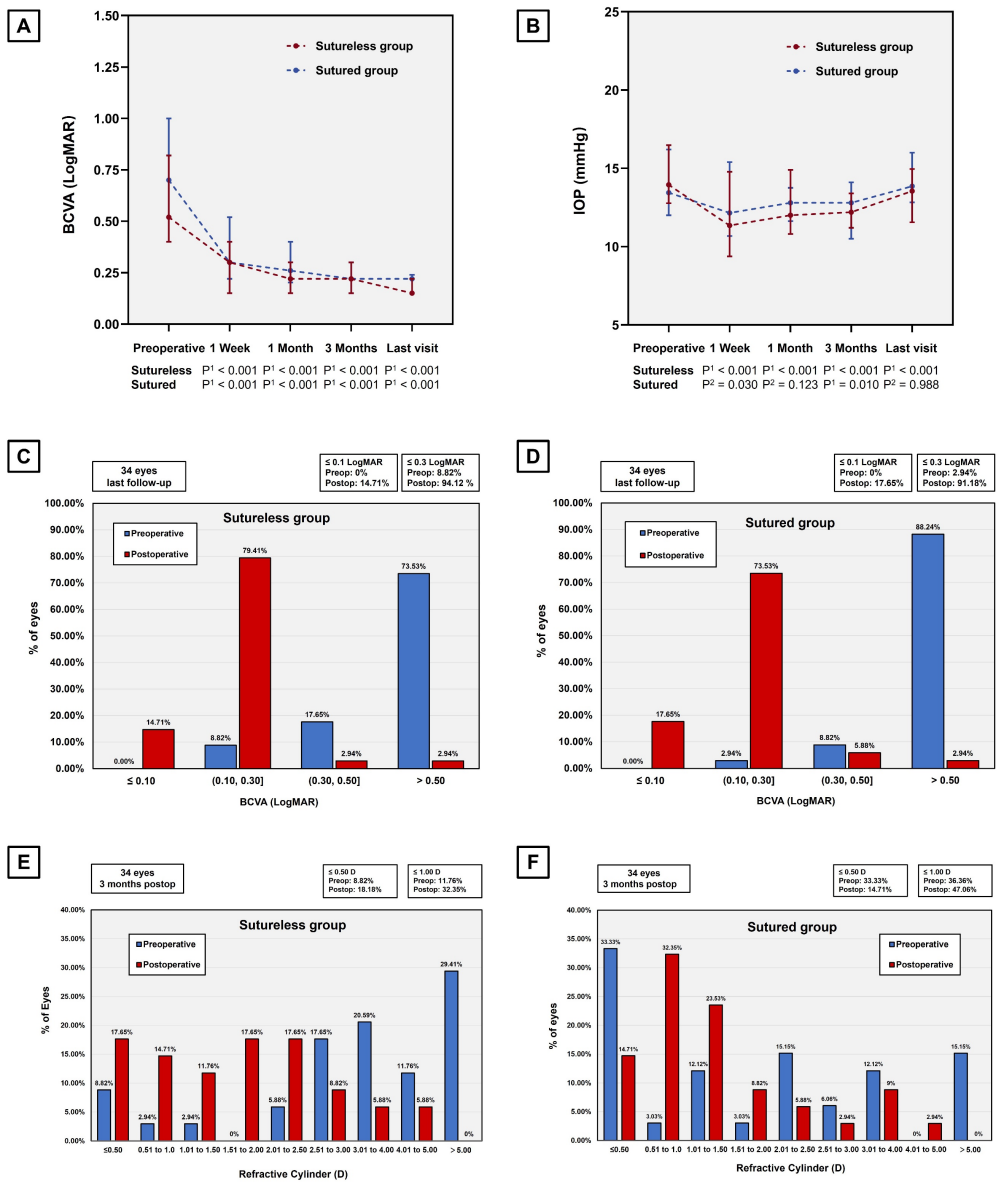
Visual and refractive outcomes between the sutureless group and the sutured group. (A) Changes in BCVA from baseline to different follow-up visit postoperatively. The temporal dynamic of BCVA in eyes at different time points (1 week, 1 month and 3 months) postoperatively indicated a significant improvement after surgery. (P, Preoperative vs. Postoperative; P^1^, Wilcoxon signed rank test; P^2^, paired Student's t-test.). (B) Changes in IOP from baseline to different follow-up visit (1 week, 1 month and 3 months) postoperatively. (P, Preoperative vs. Postoperative; P^1^, Wilcoxon signed rank test; P^2^, paired Student's t-test.). (C&D) The changes in proportion of the eyes with BCVA ≤ 0.10, 0.10-0.30, 0.30-0.50 and > 0.50 preoperatively versus final follow-up postoperatively show significant improvement in both groups. (E&F) Stacked histogram showing the percentage of eyes with manifest refractive cylinder at different thresholds before and 3 months after surgery in both groups. BCVA, best-corrected visual acuity; IOP, intraocular pressure; LogMAR, logarithm of the minimal angle of resolution.

A comparison of visual and refractive outcomes between the sutureless and sutured group was shown in Table 2. There were no statistically significant differences in postoperative UCVA and BCVA between the suturelss group and the sutured group 3 months after surgery. As shown in Figure 2 A, most of the eyes showed improved BCVA, and the difference was significant in both the sutureless group and the sutured group (P < 0.001). At last follow-up appointment, the proportion of eyes with BCVA 0.3 logMAR were higher in the sutureless group (94.12%) than that in the sutured group (91.18%), while no significant differences were detected between two groups (Figure 2 C&D). Both of the procedures did lower the IOP (Sutureless group, P < 0.001; Sutured group, P = 0.010), but the postoperative IOP is similar between two groups 3 months postoperatively (Figure 2B). Regarding the refractive outcomes, no significant difference was observed in postoperative manifest refraction spherical equivalent, manifest refraction sphere and cylinder between groups with sutured and sutureless wound closure. On analyzing manifest refractive cylinder at different thresholds, we collected the histograms of the refractive cylinder before and after surgery in the sutureless and sutured groups as shown in Figure 2 (E&F). At 3 months following the procedure, a total of 18.18% and 14.71% of eyes had astigmatism of ≤ 0.50 D in the sutureless and the sutured group, respectively. There was no statistically significant difference in the percentage of eyes within any of the manifest refractive cylinder thresholds between the two groups.

### Corneal astigmatism and surgically induced astigmatism in the sutureless and sutured group

**Figure 3:**
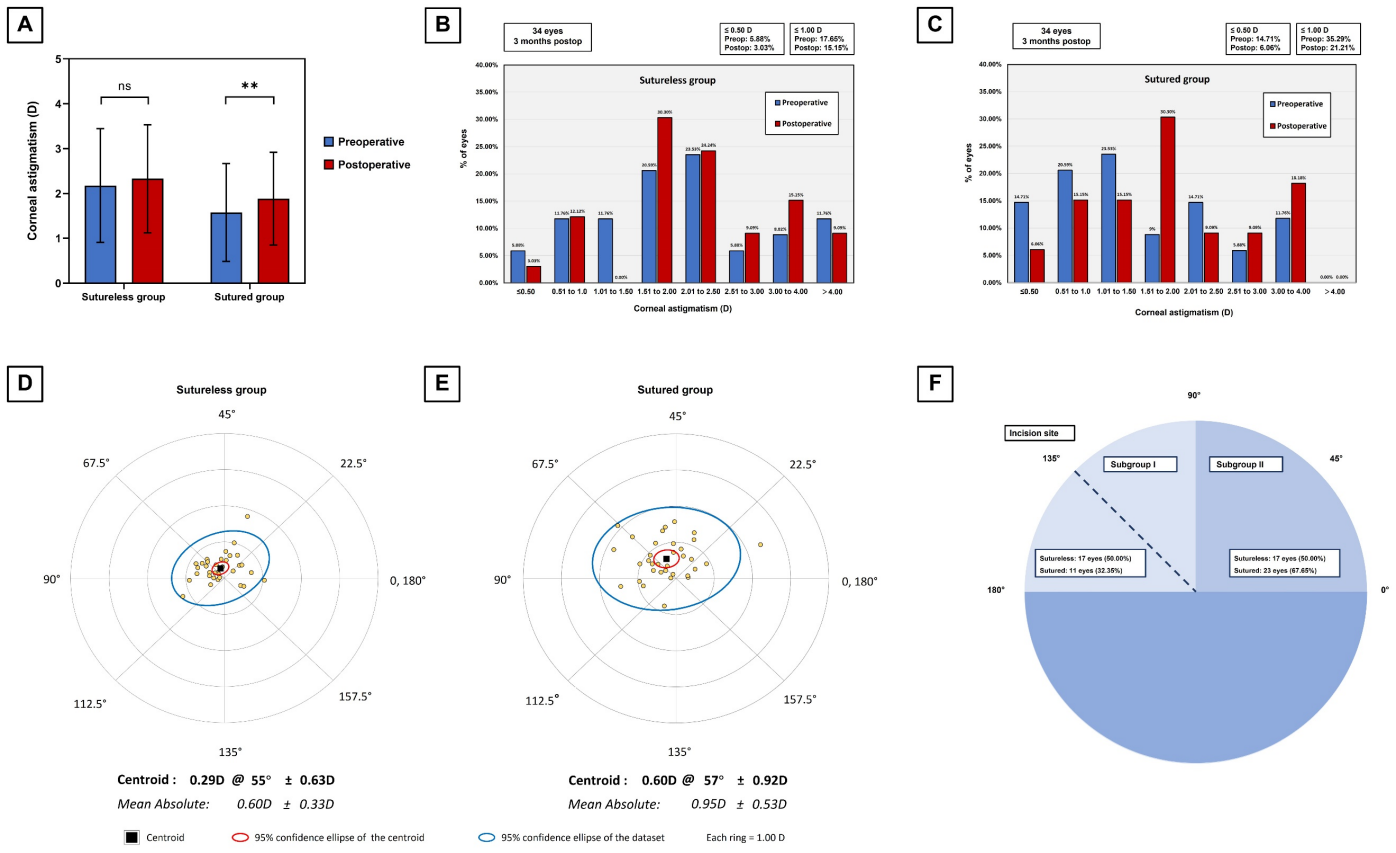
Corneal astigmatism and surgically induced astigmatism between the sutureless group and the sutured group. (A) Graph showing the magnitude of corneal astigmatism preoperatively and 3 months postoperatively in the sutureless and sutured groups. The bar represents standard deviation. D, diopters; “ns” indicates no statistically significant difference; ** Indicates statistically significant difference (** P < 0.01). (B&C) Stacked histogram showing the percentage of eyes with corneal astigmatism at different thresholds before and 3 months after surgery in both groups. (D&E) Double angle plots of the individual surgically induced astigmatism in the sutureless and sutured groups. (F) Schematic diagram of two subgroups according to the differences between the preoperative astigmatism axis (flattest axis) and the incision axis (135 degrees). Subgroup I, 0 ≤ absolute value of the difference ≤ 45 degrees; Subgroup II, 45 < absolute value of the difference.

The preoperative and postoperative corneal astigmatism in magnitude and the histogram of the corneal astigmatism in the sutureless and sutured group were shown in Figure 3 (A-C). The magnitude of corneal astigmatism was significantly increased from 1.42 D (IQ: 0.65, 2.20) preoperatively to 1.63 D (IQ: 1,05, 2.70) postoperatively in the sutured group (Wilcoxon paired signed rank test, P = 0.002), but not significantly increased from 2.03 D (IQ: 1.46, 2.62) preoperatively to 2.20 D (IQ: 1.63, 2.97) postoperatively in the sutureless group (Wilcoxon paired signed rank test, P = 0.061). The double angle plots for displaying individual SIA distributions were presented in Figure 3 (D&E). The C-SIA was 0.29 ± 0.63 D at an axis of 55° in the sutureless group and 0.60 ± 0.92 D at an axis of 57° in the sutured group. A significant lower in the magnitude of SIA (Mann-Whitney test, P = 0.001) was observed in the sutureless group (Median: 0.57, IQ: 0.39, 0.73) than in the sutured group (Median: 0.88, IQ: 0.63, 1.35), and the difference was significant after adjusting for correlation between eyes by generalized estimated equation model (P = 0.001). Furthermore, we performed subgroup analysis according to the preoperative astigmatism axis (preoperative astigmatism axis-stratified group) to further investigate the SIA in relation to the location of corneal incision (Figure 3F).

In the subgroup I (0 ≤ absolute value of the difference ≤ 45 degrees), the magnitude of SIA did not differ significantly (Mann-Whitney test, P = 0.832) between the two groups, whereas the C-SIA was higher in the sutured group (0.51 D @ 41° ± 0.70 D) than in the sutureless group (0.16 D @ 54° ± 0.95 D) (Supplementary Figure 1 A&C). In the subgroup II (45 < absolute value of the difference), the magnitude of SIA was significantly (Student's t-test, P < 0.001) lower in the sutureless group than in the sutured group, and the C-SIA was also lower in the sutureless group (0.32 D @ 61° ± 0.60 D) than in the sutured group (0.69 D @ 63° ± 0.99 D) (Supplementary Figure 1 B&D).

### Complications and details of cost for suture removal in the sutured group

No cases of intraoperative complications including posterior capsule rupture, radial tears in capsulorhexis, vitreous loss, iris prolapse, and postoperative complications including endophthalmitis, retinal detachment, and dislocation of IOL-capsular bag complex were observed in both groups. Suture-related complications included loose suture with discomfort in 5 (14.71%) eyes, loose suture with mucus infiltration in 3 (8.82%) eyes in the sutured group. There was no eye developing inflammatory reaction with corneal neovascularization and conjunctival hyperemia. In total, 22 sutures (64.71%) of 34 eyes required removal, including 16 removed along with the surgery of the second eye, and 6 under repeat general anesthesia. For the remaining 10 eyes (35.71%) with sutures that developed no suture-related complications, close follow-ups were scheduled for all these patients. No patient suffered postoperative complications such as endophthalmitis after suture removal. Among the 5 patients who underwent additional surgery for suture removal, the total cost of examinations (laboratory and imaging tests) ranged from US $153.58 up to $196.45 and the total cost of hospitalization ranged from $462.78 up to $662.87. Details of costs were provided in Supplementary Table 1.

## Discussion

The advancement in surgical techniques for CEL patients has transitioned from larger incisions to micro-incisions, aided by the introduction of intrascleral fixation of CH utilizing a 5-0 polypropylene thread. Throughout these surgeries, incision closure is achieved through suturing. Despite being regarded as the gold standard for incision closure in pediatric lens surgeries, suture application can lead to various complications including loose sutures, breaking of sutures, astigmatism, accumulation of mucus, vascularization.^19^, ^20^ Although studies have shown favorable outcomes in pediatric surgeries with sutureless wound closures to avoid suture-related complications^10^-^12^, similar investigations have not been undertaken in the patients with CEL. For the surgical management of CEL, methods such as transscleral suture-fixated IOL implantation and MCTR implantation have been widely adopted, often requiring corneal sutures due to their intricate procedures and larger incisions (greater than or equal to 2.6 mm). In contrast, the intrascleral fixation of CH represents an improvement in sutureless surgical technique, involving relatively smaller incision (2.0 - 2.2 mm), shortened operation time, better outcomes, and a lower risk of complications. This advancement provides favorable conditions for sutureless wound closure. In light of these considerations, we propose that attempting sutureless corneal incision in CEL patients who have undergone phacoemulsification combined with CTR and CH, along with in-the-bag IOL implantation, represents a significant endeavor.

**Figure 4:**
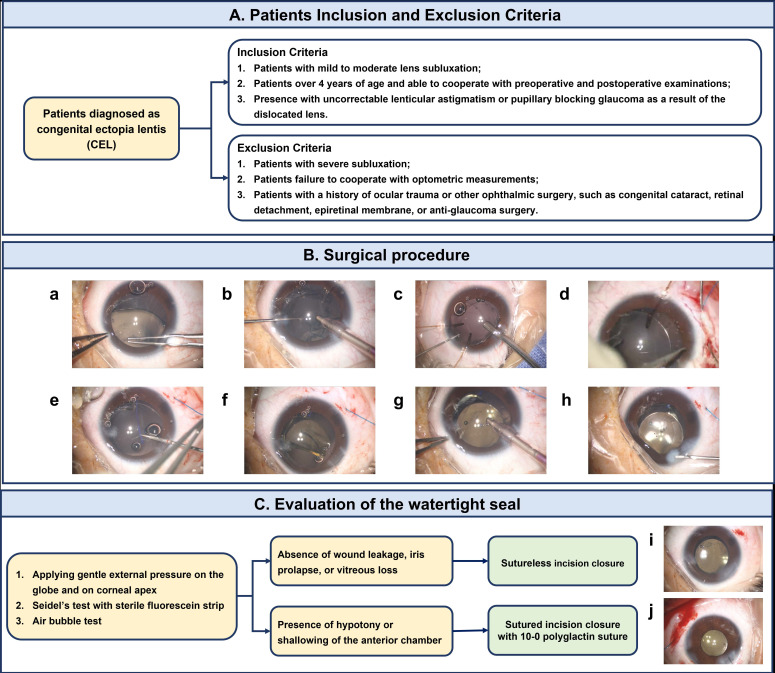
Patient eligibility criteria, surgical procedure and evaluation of the watertight seal. (A) The inclusion and exclusion criteria for patients diagnosed as CEL. (B) The surgical procedure of phacoemulsification combined with intrascleral fixation of CH and implantation of CTR and in-the-bag IOL in CEL patients. (a) Continuous circular capsulorhexis with a diameter of 4.0-5.0 mm was performed carefully. (b) The lens was removed using irrigation/aspiration mode at low vacuum, slow aspiration flow rate, and reduced bottle height, with the aid of capsular hooks. (c) The pre-loaded CTR was implanted into the capsular bag. (d) The CH was made of 5-0 commodity polypropylene thread and the needle attached to the capsule hook was inserted through the main or side-point incision and passed around the interlamellar sclera with a modified knotless Z-suture technique. (e) The externalized shaft of the hook was held and pulled by the needle holder or forceps to adjust its tension. (f) The pre-loaded IOL was injected into the anterior chamber through a 2.2-mm clear corneal tunnel incision. (g) The corneal wound was closed by stromal hydration. (C) A flowchart of the evaluation of the watertight seal and wound leakage. (i) Sutureless corneal incision. (j) Sutured corneal incision. CEL, congenital ectopia lentis; CH, capsular hook; CTR, capsular tension ring; IOL, intraocular lens.

The detailed processes in our study are shown in Figure 4 and the decision to close the wound with or without sutures is based on the following factors. First, only the children who are older than 4 years old and able to cooperate for postoperative examinations can be eligible for inclusion. Besides, the degree of lens subluxation should be mild and moderate, which is the surgical indication for intrascleral fixation of CH in CEL patients. Theoretically, with the assistance of CTR to stabilize the capsular bag, one or two hooks are adequate to support the capsular bag with zonulodialysis of less than 8 clock hours. In zonulodialysis of more than 8 clock hours, this technique is not recommended because the extensive relaxation of the zonular support substantially limits the maneuverability of managing the lens (capsulorhexis, nucleus, and cortex removal) with a floppy capsular bag.^21^ During the surgery, none of the intraoperative complications are encountered, including iris prolapse, vitreous loss and posterior capsule rupture. After creating a watertight closure, the stability of anterior chamber and intraocular pressure should be evaluated carefully and the watertightness is assessed by applying gentle external pressure on corneal apex. Following Sen *et al.*'s approach, Seidel's test with sterile fluorescein strip and air bubble tests are employed to examine the wound leakage. 12 In the absence of wound leakage indications, such as hypotony or shallowing of the anterior chamber, corneal wounds remained sutureless. Otherwise, sutures were utilized to close the incision in case of any leakage signs.

To investigate the effect of sutureless surgery in CEL patients, we conducted a comparative analysis of clinical outcomes between groups with sutured and sutureless wound closure. No significant differences in visual changes were identified between the sutureless and the sutured groups, being consistent with the study reported by Broyles *et al.* evaluating the visual outcomes in pediatric cataract surgery with or without corneal sutures.^11^ This similarity in outcomes could be attributed to advancements in surgical techniques leading to improved clinical outcomes for CEL patients. In our series, both sutureless and sutured wound closure groups exhibited comparable results in terms of visual acuity. At the last follow-up, 94.12% and 91.18% of eyes in the sutureless and sutured groups, respectively, achieved a BCVA of 0.3 logMAR or better. These outcomes are consistent with previous studies involving children with CEL who underwent MCTR implantation, where reported rates ranged from 94.7% to 98%.^6^, ^22^ In addition, there were no instances of leakage observed in the sutureless group post-surgery, and the postoperative IOP remained comparable to that of the sutured group. With regard to the refractive results, there were no significant differences between the two groups. The percentage of eyes with postoperative cylinder within ± 0.50 D was comparable, albeit slightly higher in the sutureless group (18.18%) compared to the sutured group (14.71%). These findings have demonstrated a satisfactory result of sutureless corneal incision, comparable to that of sutured corneal incision in terms of the visual and refractive outcomes.

Regarding changes in corneal astigmatism, a vector analysis has been performed to provide a more comprehensive evaluation of astigmatic alterations. The magnitude of corneal astigmatism increased in both groups, while no significant difference in the postoperative corneal astigmatism between the two groups. Notably, the magnitude of the SIA was significantly smaller in the sutureless group compared to the sutured group, suggesting that the SIA induced by the sutured corneal incision was larger than that by the sutureless corneal incision due to the mechanical strength of the sutures. In addition, the surgical procedure induced the M-SIA by ~0.7 D in the sutureless group and ~0.9 D in the sutured group, which was 0.2-0.4 D larger than the M-SIA resulting from standard cataract surgery.^23^ We speculated that the primary reason for this may be the different corneal biometric characteristics in CEL patients compared with cataract patients. Consistent with prior observations in Marfan syndrome, higher corneal astigmatism and decreased corneal curvature were identified in CEL patients compared to normal subjects, which were associated with higher M-SIA in CEL patients.^24^-^26^ Given the influence of incision site on astigmatic effects, we classified preoperative astigmatism into two subgroups based on the absolute difference between preoperative astigmatism axis (flattest axis) and incision axis (135 degrees). Remarkably, in the subgroup where the incision axis lay within 45 degrees of the flattest meridian, the magnitude of SIA exhibited similarity between the sutureless and sutured groups. Conversely, in the subgroup where the incision axis exceeded 45 degrees of the flattest meridian (within the 45 degrees of the steepest meridian), the sutureless group demonstrated a significantly reduced magnitude of SIA compared to the sutured group. These findings suggested that SIA was more susceptible to the sutured wounds in locations with steep meridian axis than in those with flat meridian axis. C-SIA has been conducive to understanding the overall trend of SIA due to the higher variations in astigmatism in magnitude and direction.^27^ In our study, the magnitude of the C-SIA was far smaller in patients without corneal sutures than in those with, indicating that sutureless wound closure contributed to the reduction of SIA in CEL patients.

Although no severe adverse events of endophthalmitis, retinal detachment, iris prolapse, and dislocation of IOL-capsular bag complex took place postoperatively in both groups, we did observe suture-related complications including loose suture, accumulation of mucus and infiltration in eyes with sutured corneal wound. Barthelomew *et al.* have demonstrated a tendency for the sutures to hydrolyze earlier in child with more congestion.^28^, ^29^ This would be of concern in surgical procedures for CEL patients which tend to develop more postoperative reaction and complications. As previously reported by Broyles et al, patients also displayed greater comfort and reduced inclination to rub the operated eye in the absence of sutures post-surgery.^11^ Matalia *et al.* further concluded that loose sutures and vascularization were common factors necessitating suture removal.^30^ In our series, 7 sutures were noted as loose that needed removing. Among them, 3 eyes experienced loose sutures with mucus and were treated with antibiotic eyedrops before suture removal to prevent infection. Suture removal was performed in 13 of 28 eyes along with the surgery of the second eye, and 5 patients necessitate additional surgery under general anesthesia to remove the corneal sutures. It's noteworthy that CEL often manifests in conjunction with hereditary systemic conditions like Marfan syndrome, Weill-Marchesani syndrome, sulfite oxidase deficiency, and homocystinuria, which may complicate general anesthesia. 23, 31-33 Additionally, suture removal has been reported to be associated with a small risk of endophthalmitis. Hence, the adoption of sutureless wound closure could eliminate the need for the general anesthesia that is usually necessary for suture removal in children.

In addition to potential complications, the necessity of suture removal in pediatric patients resulted in additional hospital readmissions and increased overall expenses. In our study, the financial implications of reoperation under general anesthesia for four patients encompassed preoperative assessments (laboratory and imaging tests), costs of drugs and supplies, and various hospitalization charges. The total expenditure ranged from US $616.08 up to $796.43. In terms of cost, the sutureless wound closure is helpful to reduce the medical expenditure associated with the surgery for suture removal.

The limitation of the study included the limited duration of follow-up. Given the limited patient pool, both eyes of certain patients were included in the analysis. To address the interdependence between paired eyes within individuals, a regression analysis employing generalized estimating equations was executed. However, considering the rarity of this disease, this is the first study that has focused on the corneal sutures in surgical management of CEL patients. Further studies specifically investigating the long-term curative effects, late-onset complications and the cost-effectiveness between groups with sutured and sutureless wound closure approaches are warranted.

## Conclusions

In conclusion, our findings have demonstrated a satisfactory result of sutureless corneal incision, comparable to that of sutured corneal incision in terms of the efficacy and safety. Placing a corneal suture could increase the incidence of suture-related complications including surgically induced astigmatism, require additional surgery under general anesthesia for suture removal which adds extra cost, and likely lead to patient discomfort to the patient's experience. From our study, sutureless corneal wound appears to be a safe alternative for wound closure in surgical management for CEL patients.

## Supplementary Material

Supplementary figure and table.

## Figures and Tables

**Table 1:** Baseline characteristics and preoperative biometrics of the patients in the sutureless and the sutured group

	Sutureless group	Sutured group	P* value
Patients, n	21	21	
Number of bilateral cataract surgeries	13 (13/21)	13 (13/21)	
Gender			0.753 ^c^
Male	12	13	
Female	9	8	
Operated eyes	34	34	0.145 ^c^
Right	17	22	
Left	17	11	
Age at the surgery, years	5 (5, 8)	5 (4, 6)	0.124 ^b^
Follow up (months)	10.82 ± 4.78	10.47 ± 6.43	0.798 ^a^
IOL type			0.203 ^c^
AcrySof SN60WF (Alcon)	30	26	
TECNIS PCB00 (J&J)	4	8	
AL, mm	23.71 (23.09, 24.66)	23.50 (21.64, 25.22)	0.598 ^d^
ACD, mm	3.36 (3.22, 3.54)	3.09 (2.80, 3.42)	0.037 ^d, #^
LT, mm	3.73 (3.58, 3.94)	3.91 (3.56, 4.24)	0.185 ^d^
WTW, mm	12.00 (12.00, 12.50)	11.95 (11.60, 12.20)	0.046 ^d, #^
Km, diopters	40.26 (39.69, 41.39)	41.05 (39.35, 42.37)	0.686 ^d^
Kf, diopters	39.47 ± 1.44	40.14 ± 1.85	0.425 ^d^
Ks, diopters	41.65 ± 1.57	41.72 ± 2.02	0.913 ^d^

IOL = intraocular lens, J&J = Johnson & Johnson, New Jersey, USA; Alcon = Alcon Laboratories Inc., Texas, USA; AL = axial length; ACD = anterior chamber depth, as measured from corneal epithelium to lens; LT = lens thickness; WTW = white to white; K = keratometry; Km = mean keratometry; Kf = flat keratometry; Ks = steep keratometry. Normally distributed data was shown in the mean ± standard deviation, and was shown in median (interquartile) otherwise. * Sutureless group vs. Sutured group a. Independent samples t-test; b. Mann-Whitney test; c. Chi-square test; d. Generalized estimated equation model with adjustment of correlation between 2 eyes within same person. ^#^Statistically significant (P < .05).

**Table 2:** Comparison of visual and refractive outcomes and refractive outcomes at month 3 visits between the sutureless and the sutured group

	**Sutureless group**	**Sutured group**	P* value	P* value
**Visual outcomes**				
**UDVA, LogMAR**				
Preoperative	1.00 (0.92, 1.22)	1.00 (0.92, 1.30)	0.856 ^b^	0.839 ^c^
Postoperative (month 3)	0.40 (0.30, 0.63)	0.60 (0.35, 0.82)	0.299 ^b^	0.356 ^c^
P** value	< 0.001 ^d, #^	< 0.001 ^d, #^		
				
**BCVA, LogMAR**				
Preoperative	0.52 (0.40, 0.82)	0.70 (0.52, 1.00)	0.070 ^b^	0.328 ^c^
Postoperative (month 3)	0.22 (0.15, 0.30)	0.22 (0.15, 0.30)	0.303 ^b^	0.425 ^c^
				
**IOP, mmHg**				
Preoperative	13.95 (12.78, 16.48)	13.45 (12.00, 16.20)	0.370 ^b^	0.365 ^c^
Postoperative (month 3)	12.20 (11.20, 13.40)	12.80 (10.50, 14.10)	0.997 ^b^	0.905 ^c^
				
**Refractive outcomes**				
**Manifest refraction spherical equivalent, diopters**				
Preoperative	-8.32 (-11.63, -4.94)	-6.30 (-10.03, -0.90)	0.162 ^b^	0.054 ^c^
Postoperative (month 3)	0.57 (-1.69, 1.38)	-0.69 (-1.63, 1.56)	0.806 ^b^	0.565 ^c^
P** value	< 0.001 ^d, #^	0.002 ^d, #^		
				
**Manifest sphere, diopters**				
Preoperative	-6.38 (-9.81, -3.19)	-5.00 (-9.38, -1.13)	0.334 ^b^	0.187 ^c^
Postoperative (month 3)	1.38 (-0.88, 2.50)	0.63 (-0.81, 2.25)	0.468 ^b^	0.407 ^c^
P** value	< 0.001 ^d, #^	0.002 ^d, #^		
				
**Manifest cylinder, diopters**				
Preoperative	-4.00 (-5.63, -2.94)	-2.00 (-3.50, -0.50)	0.001 ^b, #^	0.001 ^c, #^
Postoperative (month 3)	-1.75 (-2.50, -1.00)	-1.25 (-2.00, -0.75)	0.198 ^b^	0.340 ^c^
P** value	< 0.001 ^d, #^	0.191 ^d^		

UCVA, uncorrected distance visual acuity; BCVA, best-corrected visual acuity; LogMAR, logarithm of the minimal angle of resolution; IOP, intraocular pressure. Normally distributed data was shown in the mean ± standard deviation, and was shown in median (interquartile) otherwise. * Sutureless group vs. Sutured group, ** Preoperative vs. Postoperative. a. Independent samples t-test; b. Mann-Whitney test; c. Generalized estimated equation model with adjustment of correlation between 2 eyes within same person; d. Wilcoxon signed rank test. ^#^Statistically significant (P < .05).

**Table 3:** Comparison of postoperative surgically induced astigmatism between the sutureless and the sutured group

	Sutureless group	Sutured group	P* value	P* value
**SIA, diopters**	0.57 (0.39, 0.73)	0.88 (0.63, 1.35)	0.001 ^b, #^	0.001 ^c, #^
Subgroup I	0.59 (0.34, 0.78)	0.67 (0.34, 0.96)	0.520 ^b^	0.832 ^c,^
Subgroup II	0.59 ± 0.26	1.07 ± 0.54	0.002 ^a, #^	<0.001 ^c, #^

SIA = surgically induced astigmatism; ^#^Statistically significant (P < .05). Normally distributed data was shown in the mean ± standard deviation, and was shown in median (interquartile) otherwise. * Sutureless group vs. Sutured group. a. Independent samples t-test; b. Mann-Whitney test; c. Generalized estimated equation model with adjustment of correlation between 2 eyes within same person. ^#^Statistically significant (P < .05).
