# Supplementary material for: Application of sutureless corneal incision for patients with congenital ectopia lentis - Is it feasible, effective and safe?
Source: Int J Med Sci. 2024 May 30;21(8):1541–51. doi: 10.7150/ijms.93994 (PMC11186419; doi:10.7150/ijms.93994)

Supplementary Table 1. Suture-related complications and details of cost for suture removal in the sutured group.

	Sutured group (n = 28 eyes)
**Suture-related complications, n**	
Loose suture only	5 (14.71%)
Loose suture with mucus infiltration	3 (8.82%)
Inflammatory response with vascularization	0
**Suture removal, n**	
Total sutures removed	22 (64.71%)
Sutures removed along with the surgery of the second eye	16 (72.73%)
Sutures removed repeat general anesthesia	6 (27.27%)
**Expenditures for suture removal, US$**	**Median (Range: Min, Max)**
Total cost of preoperative examinations	163.03 (153.58, 196.45)
Total cost of hospitalization	482.84 (462.78, 662.87)
Medication costs	72.25 (53.76, 119.44)
Surgical costs	18.89 (16.71, 37.78)
Anesthesia costs	168.11 (165.64, 168.11)

Supplementary Figure 1. Double angle plots of the individual surgically induced astigmatism in two subgroups between sutureless and sutured groups.

(A&B) Subgroup I and Subgroup II in the sutureless group.

(C&D) Subgroup I and Subgroup II in the sutured group.

Supplementary Figure 1

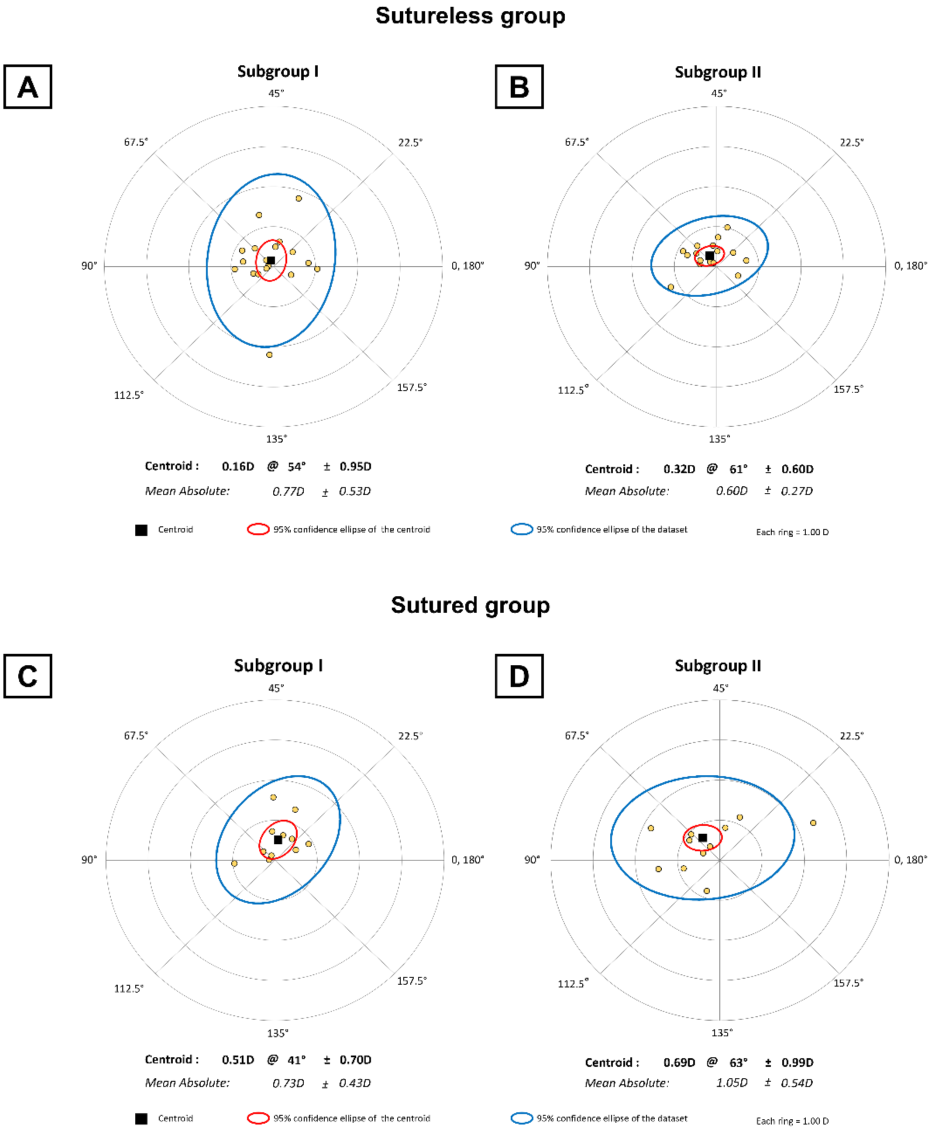

Supplement: Supplementary file 1 — Supplementary figure and table. [file ijmsv21p1541s1.pdf]
